# Imiquimod 3.75% Cream Applied Daily to Treat Anogenital Warts: Combined Results from Women in Two Randomized, Placebo-Controlled Studies

**DOI:** 10.1155/2011/806105

**Published:** 2011-08-24

**Authors:** David A. Baker, Daron G. Ferris, Mark G. Martens, Kenneth H. Fife, Stephen K. Tyring, Libby Edwards, Anita Nelson, Kevin Ault, Kenneth F. Trofatter, Tiepu Liu, Sharon Levy, Jason Wu

**Affiliations:** ^1^Division of Infectious Disease, Department of Obstetrics, Gynecology & Reproductive Medicine, Stony Brook Health Science Center, Stony Brook University Medical Center, Stony Brook, NY 11794-8091, USA; ^2^Departments of Family Medicine and Obstetrics & Gynecology, Medical College of Georgia, Augusta, GA 30912, USA; ^3^Department of Obstetrics & Gynecology, Jersey Shore University Medical Center, Neptune, NJ 07753, USA; ^4^Department of Medicine, Indiana University, Indianapolis, IN 46202, USA; ^5^Department of Dermatology, University of Texas-Houston Medical School, Houston, TX 77030, USA; ^6^Mid-Charlotte Dermatology and Research, Charlotte, NC 28211, USA; ^7^Department of Obstetrics & Gynecology, David Geffen School of Medicine, University of California, Los Angeles, CA 90095, USA; ^8^Department of Gynecology and Obstetrics, Emory University School of Medicine, Atlanta, GA 30322, USA; ^9^Department of Obstetrics and Gynecology, University of South Carolina School of Medicine at Greenville, Greenville, SC 29203, USA; ^10^Product Development, Graceway Pharmaceuticals, LLC, Exton, PA 19341, USA; ^11^Biostatistics, The Medicines Company, Parsippany, NJ 07054, USA

## Abstract

*Objective*. To evaluate if new imiquimod formulations using a shorter treatment duration are safe and efficacious to treat anogenital warts. *Methods*. In two studies 534 women ≥12 years of age (mean 33.4) with 2–30 warts (mean 7.9) and total wart area ≥10 mm^2^ (mean 166.3) were randomized (1 : 2 : 2) to placebo (106), imiquimod 2.5% (212) or 3.75% (216) creams applied once daily until complete clearance or a maximum of 8 weeks. *Results*. For placebo, imiquimod 2.5% and 3.75%, respectively, complete clearance of all warts was achieved in 14.2%, 28.3%, and 36.6% of women (intent-to-treat, *P* = 0.008 imiquimod 2.5%, and *P* < 0.001 3.75% versus placebo). Mean changes in wart counts were −10.7%, −50.9%, and −63.5% (per-protocol, *P* < 0.001 each active versus placebo) and safety-related discontinuation rates 0.9%, 1.4%, and 2.3%. *Conclusions*. Imiquimod 3.75% applied daily for up to 8 weeks was well tolerated and superior to placebo in treating women with external anogenital warts.

## 1. Introduction

Human papillomavirus (HPV) infection is the most frequent sexually transmitted disease in the United States [1]. The major clinical focus in women with HPV has been on the prevention of cervical cancer, predominantly associated with oncogenic HPV types such as 16 and 18, through screening, and, more recently, through HPV vaccination [2]. External (ano)genital warts (EGW), however, are also a common manifestation of  HPV infection, usually associated with HPV types 6 and 11, types not included in one of the two marketed vaccines [3]. EGWs are frequently multicentric and recurrent. The estimated prevalence of EGWs in the sexually active population between 15 and 49 years of age in the United States is 1% [4]. Up to 7.2% of women report a history of EGWs [5]. A significant detrimental impact on quality of life was observed in adults with a current diagnosis of EGWs, particularly in young women [6]. In a study assessing the psychosocial burden of HPV-related disease, women with EGWs reported lower general quality of life scores than women with abnormal Papanicolaou smears or biopsy-proven cervical intraepithelial neoplasia [7]. With an HPV-specific impact profile instrument, the impact on quality of life of having EGWs was second to that of having cervical intraepithelial neoplasia 2/3 [7]. EGWs also represent a significant burden to the health care system with an estimated 385,000 initial visits to physician offices in 2008 [8] and $200 million in direct costs annually in the United States [9]. 

Treatments for EGWs focus on the removal of visible warts [3] and can be divided broadly into two categories: provider-administered ablative/cytodestructive therapies (including cryotherapy, laser ablation, and trichloroacetic acid) and patient-administered topical therapies (such as podophyllotoxin, sinecatechins, and imiquimod) [10]. Imiquimod directly activates innate immune cells through Toll-like receptor 7, resulting in production of cytokines [11]. Indirectly, imiquimod enhances antigen-specific cell-mediated immunity [11]. Imiquimod 5% cream was approved in 1997 to treat EGWs using a regimen of application to warts 3 times per week (3x/week) until complete clearance of baseline/target warts in the designated anogenital area(s) or for a maximum of 16 weeks of treatment [12]. Long treatment durations are one of the impediments to treatment adherence [13–15]. While increasing the dosing frequency might be considered to shorten the treatment duration with imiquimod, application of imiquimod 5% more frequently than 3x/week resulted in a greater incidence and severity of local adverse events, without an improvement in efficacy [16–18].

Formulations with lower concentrations of imiquimod were developed to potentially allow for a decrease in treatment duration by using daily dosing. Two identical placebo-controlled phase 3 studies have been recently completed evaluating the safety and efficacy of imiquimod 2.5% and 3.75% cream applied once daily for up to a maximum of 8 weeks to treat EGWs in women and men [19]. As gender stratification in randomization was included in the design of the studies, and the safety and efficacy outcomes differed between women and men [19], we herein report the results from the women enrolled in the studies; the results from men will be reported separately.

## 2. Methods

### 2.1. Study Population

Women participating in the studies were 12 years or older, in general good health, with 2 to 30 EGWs in the vulvar (including mons), inguinal, perineum, and/or perianal areas, and with a minimum total wart area of 10 mm^2^. EGWs were diagnosed clinically; to mimic real world practice, histologic confirmation was not required. Exclusions included known human immunodeficiency virus infection, immunosuppression, other genital infections, allergy to imiquimod or cream excipients, history of high-risk type HPV infection, high-grade pathology on Papanicolaou smear, pregnancy, or lactation. Additional exclusions included imiquimod or HPV vaccination within 1 year, and sinecatechins within 12 weeks, cytotoxics, immunomodulators/immunosuppressives, systemic antivirals (excluding oral antiherpes agents and oseltamivir), investigational therapies, and any treatments procedures within the anogenital area within 4 weeks. Women agreed to refrain from sexual activity while the study drug was on their skin. Women also agreed to use adequate contraception during the study.

The studies were conducted in compliance with Good Clinical Practice guidelines and approved by a central institutional review board; at some study centers, approval was also obtained from a local institutional review board. All participants provided written informed consent. For women <18 years of age, consent was obtained from the parent or legal guardian and assent from the minor. Enrollment began in June 2008, and all study procedures were completed by June 2009. Each of the two studies was registered at www.clinicaltrials.gov (NCT00674739 and NCT00735462).

### 2.2. Study Designs and Study Drug Dosing

Women were enrolled at 70 study centers in the United States. Each study center participated in only one of the two studies. Each study included a screening visit, an evaluation phase (treatment period of up to 8 weeks and a no-treatment period of up to 8 weeks) of up to 16 weeks, and an observational follow-up phase of up to 12 additional weeks in women with complete clearance. A no-treatment period was included to allow adequate resolution of local skin reactions (LSRs) or application site reactions (ASRs) that might interfere with wart assessments as well as to determine if residual warts would resolve without further treatment. An observational follow-up phase of 12 weeks was included to assess sustained complete clearance in those women who achieved initial complete clearance.

Identically appearing study kits were prepackaged for each study center according to a computer-generated randomization schedule using a 1 : 1 : 2 allocation for placebo, imiquimod 2.5%, and imiquimod 3.75% cream (3 M Healthcare, Loughborough, UK) and a block size of 5. Eligible women were randomized at each study center by assigning them to study kits in sequential order. The treatment assignment was concealed from the participant, the investigators and their staff, and the clinical research team.

Participants were instructed to self-apply up to one packet (250 mg cream) of study drug once daily to warts identified at baseline as well as to any new warts that developed during the treatment period. A thin layer of the cream was applied to cover each wart area, prior to normal sleeping hours, and removed approximately 8 hours later by washing. Women were told not to apply study cream to the urethra, vagina, or cervix. Internal warts, for example, vaginal or cervical, were neither treated nor counted as EGWs. Temporary dosing interruptions (rest periods) were allowed to manage an LSR or an adverse event (AE); treatment was resumed upon adequate resolution. Missed doses were not to be made up, and the maximum duration of treatment was 8 weeks (including rest periods). Participants were assessed every 2 weeks during the evaluation phase. Participants with complete clearance of all warts within all anogenital areas entered the 12-week observational follow-up period and were assessed every 4 weeks or until they had a “recurrence” of any wart (baseline or new) in any anogenital area.

### 2.3. Efficacy Evaluation

The primary assessment was based on the count of all EGWs (baseline and new, treated or untreated) in all anogenital areas. A cluster of warts was counted as a single wart. The primary efficacy endpoint for each study was the complete clearance rate, defined as the proportion of participants by the end of study (EOS) visit with a zero count of EGWs in all of the anogenital anatomic areas. Key secondary endpoints included the partial clearance rate (participants with ≥75% reduction in EGW count), the change in wart counts from baseline, and the 12-week sustained clearance rate. For each study, sample sizes (combined population including both genders) were selected for placebo (90) and each active group (180) to have at least a 90% power at a two-sided overall 5% level of significance, adjusting for an estimated drop-out rate of 20%, to detect a difference in complete clearance rates of 30% for the active groups versus 10% for the placebo group. Replacement of participants was not allowed. The individual studies were not prospectively powered for analysis by gender. For intent-to-treat (ITT) analyses, imputations were made for missing data points using last observation carried forward. Complete clearance rates and partial clearance rates were analyzed using Cochran-Mantel-Haenszel statistics, stratifying by center, and for the overall population analyses, by gender. Confidence intervals were calculated using exact binomial statistics. Pair-wise comparisons were performed with Hochberg's modified Bonferroni procedure [20]. The percent change in wart count was analyzed using Analysis of Covariance. Per-protocol (PP) analyses were performed on a subset of participants who met predefined criteria for protocol compliance. All statistical analyses were performed using SAS (Version 9.1.3, SAS Institute, Inc., Cary, NC). 

### 2.4. Safety Evaluations

Hematology, serum chemistry, and urinalysis tests were performed prestudy and at EOS. For women of childbearing capacity, pregnancy tests were performed at prestudy, treatment initiation, and every 4 weeks up to and including end of treatment (EOT). Spontaneously reported AEs were collected at each visit and graded as none, mild, moderate, and severe. AEs were coded using the Medical Dictionary for Regulatory Activities (Version 11.0). LSRs (erythema, edema, weeping/exudate, flaking/scaling/dryness, scabbing/crusting, and erosion/ulceration) in the anogenital locations were also assessed by the investigator at each visit. Each LSR was graded as none, mild (not applicable to erosion/ulceration), moderate or severe. LSRs were analyzed separately from other AEs as they were collected systematically.

## 3. Results

### 3.1. Subject Population

**Figure 1:**
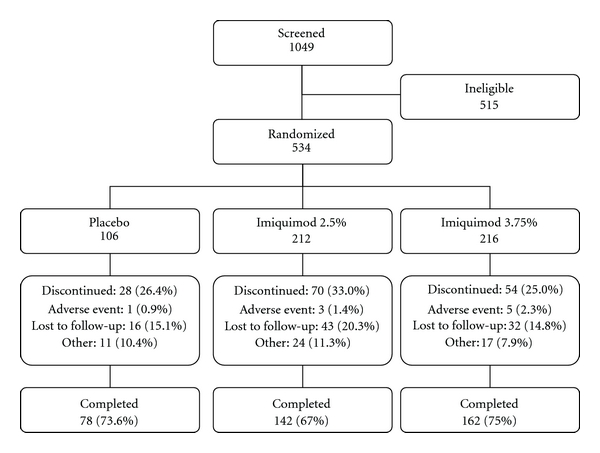
Disposition of women in the studies, combined, evaluation phase. Percents displayed are percent of women randomized by treatment group. Adverse events category includes women discontinuing for local skin reactions.

In the two studies combined, 1049 women were screened, 534 were enrolled and 382 completed the studies (Figure 1). Of the 515 women who were screen failures, the most frequent reasons for exclusion were not meeting the EGW diagnosis and wart count requirements (272, 52.8%), “other” (93, 18.1%), and not willing to comply with study requirements (49, 9.5%). The overall subject noncompletion rate was 28.5% (152 women); approximately 60% of these women (91) were lost to follow-up. Discontinuation rates were comparable across the three treatment groups (Figure 1). Of the 143 women excluded from the PP analysis, 138 were excluded for “treatment noncompliance,” most because they were lost to follow-up early in the study and did not meet the minimum criteria for treatment exposure.

Study participant characteristics by treatment group are presented in Table 1. The mean age was 33.4 years; only three women were <18 years of age. Overall, 67.4% of the women were white, the mean baseline wart count was 8.7, and the mean total wart area was 166.3 mm^2^. More than two-thirds (69.3%) of the women reported that the current episode of EGW was their first. The majority of women (54.5%) had warts on 2 or more anogenital locations; the most frequently involved locations were vulvar (64.8%) and perineal (47.9%). The duration since EGW diagnosis for the overall population was 5.6 years. There were no statistically significant differences among the treatment groups with regard to the baseline characteristics examined (Table 1).

### 3.2. Efficacy

**Figure 2:**
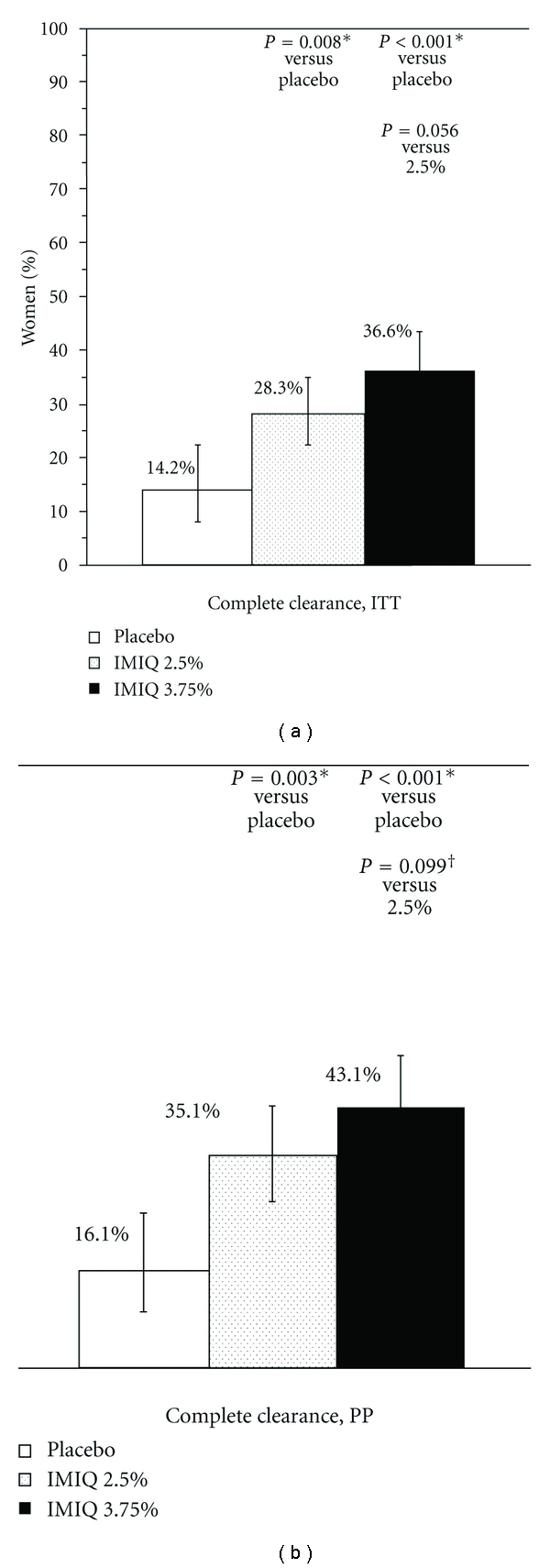
Complete clearance rates. (a) Intent to treat (b) per protocol. White: placebo, stippled: imiquimod 2.5%, solid black: imiquimod 3.75%. *P*-values from a Cochran-Mantel-Haenszel test, stratified by analysis site using two treatment groups at a time.*indicates statistically significant for active versus placebo, and ^†^for imiquimod 3.75% versus 2.5%, using Hochberg's modified Bonferroni procedure. Bars indicate 95% confidence interval calculated using exact method.

For the primary endpoint of complete clearance of all warts (baseline and new) at EOS, in the combined analyses in women, both imiquimod groups were superior to placebo (Figures 2(a), ITT, and 2(b), PP). In the individual studies, only imiquimod 3.75% was statistically superior to placebo in women in both of the studies (34.0% versus 16.0%, ITT,  *P* = 0.017; 38.8% versus 12.5%, ITT, *P* < 0.001). Imiquimod 2.5% was only superior to placebo in one study. 

For the women who achieved complete clearance in the combined analyses, median time to clearance was 71.0, 60.0, and 57.0 days for placebo, imiquimod 2.5%, and 3.75%, respectively. For imiquimod 3.75%, complete clearance was similar between women ≤35 versus >35 years, with one versus multiple anatomical sites involved, or with first episode versus nonfirst episode disease. Complete clearance rates (ITT) were numerically higher for those women who were nonwhite (28/59, 47.5%) versus white (51/157, 32.5%), had baseline wart counts ≤7 (54/133, 40.6%) versus >7 (25/83, 30.1%), had total wart area ≤150 (66/158, 41.8%) versus >150 mm^2^ (13/48, 27.1%), and had EGW diagnosis duration ≤6 months (33/64, 51.6%) versus >6 months to ≤24 months (17/55, 30.9%) and ≥24 months (29/97, 29.9%). In multivariate analyses, imiquimod 3.75% was superior to placebo in women after adjustments for age, race, baseline wart count, baseline total area, first versus nonfirst episode, and EGW duration.

**Figure 3:**
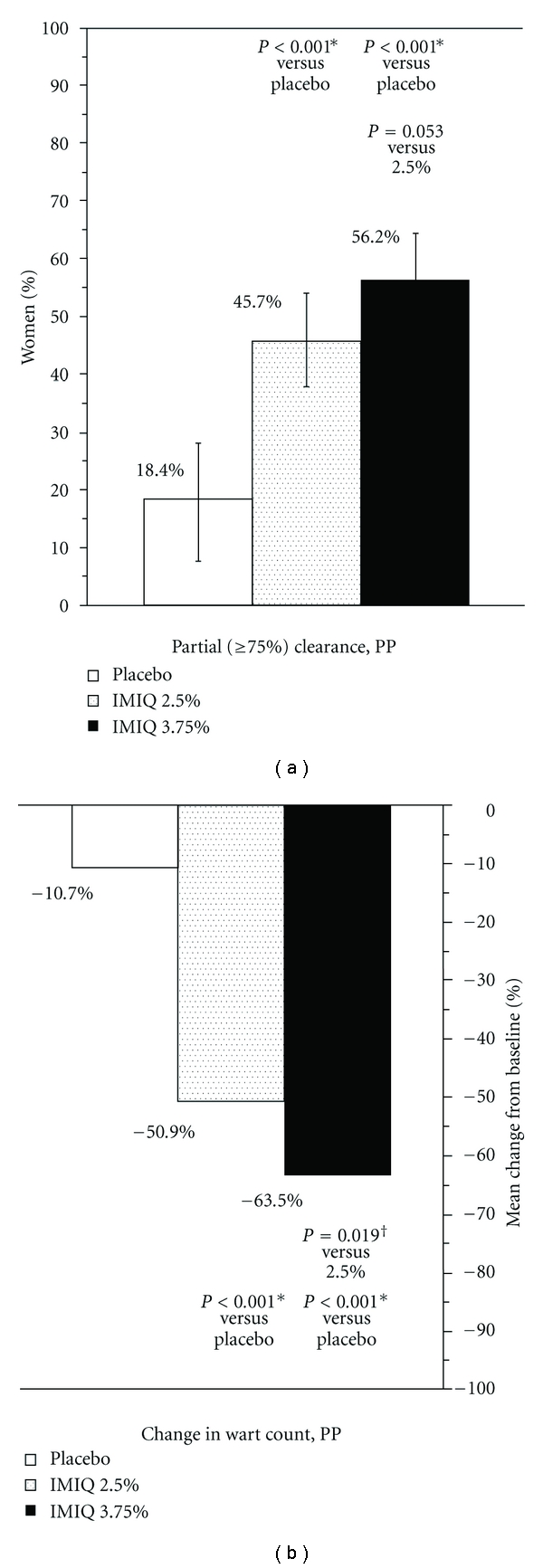
Partial clearance rates and change in wart count from baseline. White: placebo, stippled: imiquimod 2.5%, solid black: imiquimod 3.75%. (a) Partial (≥75% reduction in wart count compared to baseline) clearance rates, PP. *P* values from a Cochran-Mantel-Haenszel test, stratified by analysis site using two treatment groups at a time.*indicates statistically significant for active versus placebo, and ^†^for imiquimod 3.75% versus 2.5%, using Hochberg's modified Bonferroni procedure. Bars indicate 95% confidence interval calculated using exact method. (b) Change in wart count compared with baseline, mean percent, PP. *P* values from an analysis of covariance test, controlling for baseline wart count and analysis site.*indicates statistically significant for active versus placebo, and ^†^for imiquimod 3.75% versus 2.5%, using Hochberg's modified Bonferroni procedure.

**Figure 4:**
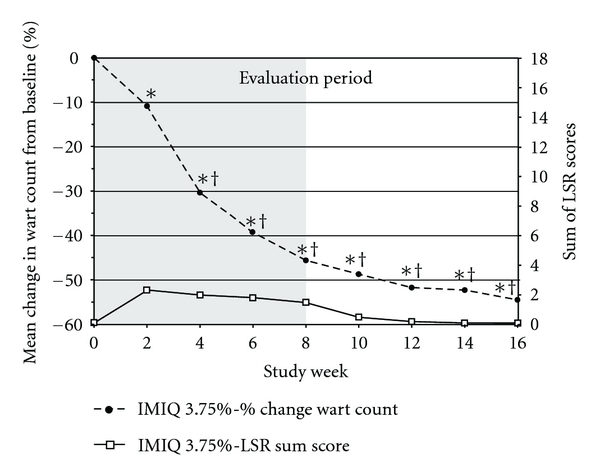
Change in wart count compared with baseline (left axis, circle, mean, ITT, last observation carried forward) versus local skin reaction (LSR) sum score (right axis, square, mean, observed) over time for imiquimod 3.75% during the evaluation phase. Maximum LSR sum score = 18. Shaded box indicates treatment period of up to 8 weeks. *P* values were calculated using analysis of covariance, controlling for baseline wart count, and analysis site.*indicates statistically significant for active versus placebo, and ^†^for imiquimod 3.75% versus 2.5%, using Hochberg's modified Bonferroni procedure.

For the secondary endpoint of partial clearance (≥75% reduction in wart count from baseline), imiquimod 3.75% and 2.5% were superior to placebo (ITT and PP; Figure 3(a) PP). Imiquimod 3.75% was also superior to imiquimod 2.5% by ITT (47.7% versus 36.3%, *P* = 0.015) but not PP. Similarly, for change (percent) in wart count from baseline, both of the imiquimod groups were superior to placebo (ITT and PP; Figure 3(b), PP). Imiquimod 3.75% was also superior to imiquimod 2.5% by ITT (mean −54.5% versus −40.1%, *P* = 0.003). A significant change in the wart count compared to baseline was observed in the imiquimod 3.75% group as early as week 2 (Figure 4, left axis).

For specific anatomic sites, imiquimod 3.75% cream was superior to placebo with respect to complete clearance for the vulvar (including mons), perineal, and perianal areas and was also superior to imiquimod 2.5% for the perianal area; complete clearance was highest for the perianal area (78.5%, Table 2, PP). Similarly, imiquimod 3.75% was superior to placebo and 2.5% for these same areas, respectively, for change in wart count compared to baseline; the mean percent change was greatest for the perianal area (−82.2%, Table 2, PP). Except for imiquimod 2.5% in the inguinal area, both imiquimod groups had higher rates of clearance and greater decreases in wart count numerically than placebo for all of the anatomical areas. The lack of statistical significance for some of the subgroup comparisons may have been due to the sample sizes.

In the women who achieved complete clearance and entered the 12-week follow-up phase, complete clearance was sustained in 9/9 (100.0%), 32/53 (60.4%), and 47/72 (65.3%) in the placebo, imiquimod 2.5%, and 3.75% groups, respectively. A recurrence, defined as any EGW in any of the anogenital locations regardless if it was baseline or new, treated or untreated, was observed in 0/9 (0%), 9/53 (17.0%), and 14/72 (19.4%) women, respectively. The rest of the women were lost to follow-up.

### 3.3. Safety

The mean numbers of days treated were 50.5 (12.0), 43.2 (15.4), and 41.4 (16.3) days for the placebo, imiquimod 2.5%, and 3.75% groups, respectively. About 40% of women in each treatment group experienced an AE (Table 3). The proportions of women with any treatment-related AE, or any ASR, were similar between the imiquimod 2.5% and 3.75% groups, but higher than in the placebo group (Table 3). Nine women (1.7%) had AEs that led them to discontinue study participation; of these, 6 (3 imiquimod 2.5%, and 3 imiquimod 3.75%) had AEs (all ASRs) considered to be related to study cream. ASRs were the AEs reported by the most women in the imiquimod groups and were the only specific treatment-related AEs reported by >1% of women overall (Table 3). The incidences of the ASRs occurring in the most (application site pain, application site irritation, and application site pruritus) were generally comparable between imiquimod 2.5% and 3.75% (Table 3).

There were 9 treatment-emergent serious AEs in 8 women; none was considered to be related to study cream. Of these serious AEs, 2 resulted in women discontinuing study participation; both were in the imiquimod 3.75% group (pelvic mass/acute abdomen and malignant melanoma). The proportion of women requiring a rest period and the mean missed doses for those who required a rest were similar for imiquimod 2.5% and 3.75% (Table 3). 

LSRs were frequent, with 69.6% and 76.8% of women in the imiquimod 2.5% and 3.75% groups, respectively, experiencing at least one LSR during the treatment phase (Table 4). The mean LSR sum score (sum of the intensities of all of the LSRs in a participant, maximum of 18) peaked early and remained relatively flat during treatment (Figure 4, right axis). Erythema and ulceration were the severe LSRs that occurred in the most women in each of the imiquimod groups; rates were slightly higher in the imiquimod 3.75% group versus the 2.5% group (Table 4). 

Clinical laboratory values, vital sign measurement and physical examinations did not raise any significant safety concerns (data not shown). Of the 8 pregnancies that occurred in the active groups, no abnormalities were reported in 3 infants; the other 5 women were lost to follow-up. There were 3 pregnancies in the placebo group.

## 4. Discussion

In each of the individual studies, imiquimod 3.75% applied daily for up to 8 weeks in women was superior (ITT) to placebo with respect to the primary endpoint of complete clearance all warts (baseline and new). These results were consistent with those for the combined male and female populations in the individual studies, where imiquimod 3.75% was also statistically superior to placebo in both studies [19].

In the combined analysis in women, imiquimod 3.75% was also superior to placebo (ITT); the complete clearance rate was higher for imiquimod 3.75% compared to imiquimod 2.5% cream, although the difference was not statistically significant. For partial clearance and change in wart count, however, imiquimod 3.75% was superior to 2.5% (ITT). Interestingly, this incremental benefit of imiquimod 3.75% versus 2.5% did not appear to be at the expense of safety. Rates of severe LSRs were slightly higher in the imiquimod 3.75% group, but overall tolerability was similar to that of imiquimod 2.5%, including the proportions of women with treatment-related AEs, who discontinued from treatment, and who required rest periods. Comparisons of safety, as well as overall safety conclusions, are limited by sample size with respect to AEs of low frequency. Qualitatively, however, the treatment-related AEs observed were consistent with the known safety profile for imiquimod 5% [21].

The combined complete clearance rates observed in women (36.6% ITT and 43.1% PP for imiquimod 3.75%) in these studies were higher than those observed in men in the same studies (18.6% ITT and 22.7% PP for imiquimod 3.75%) [19, 22]. This gender difference is consistent with prior experience with other therapies for EGWs, including imiquimod [12], sinecatechins [23], podophyllin, cryotherapy, and electrodessication [24]. Anatomic site-specific analyses in women suggest that areas with more mucosal skin (e.g., perianal areas) versus areas with more fully keratinized skin (inguinal areas) may experience better efficacy. This observation, however, is limited in that the women were not randomized based on anatomic site involvement, and other factors may be involved, such as disease severity within the anatomic site or occlusion of the skin in certain areas.

For women in the imiquimod 3.75% group who achieved complete clearance, 65.3% of these women sustained clearance during 12 weeks of observational follow-up. Sustained complete clearance rate is a higher standard than recurrence rate, which is sometimes reported. The former is what patients desire, sustained freedom from warts, while the latter may ignore untreated or new warts in the treatment area and is influenced by the lost to follow-up rate. A high sustained complete clearance rate is consistent with immune-mediated clearance induced by imiquimod and was also seen in the placebo group where complete clearance was likely due to natural development of a robust cell-mediated immune response. As very few placebo women achieved complete clearance, however, this high sustained complete clearance rate has limited clinical relevance.

The complete clearance rate observed with imiquimod 3.75% cream was lower than that reported by Edwards et al. in 1998 for women treated with imiquimod 5% cream applied 3x/week for up to 16 weeks [12]. However, there are significant limitations in making direct comparisons between the studies, because of differences in study designs, efficacy assessments, and enrolled populations. For example, in the imiquimod 5% study, biopsy confirmation was required at entry. In the current studies, biopsies were not required to be consistent with actual clinical practice; this may have diluted observed efficacy if women with conditions other than EGWs were treated. Also, biopsy itself might cause sufficient inflammation in some participants to activate the cellular immune response, confounding the results.

In the imiquimod 5% study, not all anogenital regions with warts were required to be treated (nontarget), and new warts arising during study were not included in the analyses [12]. Thus a participant could be categorized as having achieved initial “complete clearance”, as well as having “sustained complete clearance”, even if baseline “nontarget” or “new” warts were present. In contrast, to achieve initial complete clearance or have sustained complete clearance in the current studies, a woman had to have no EGWs, baseline or new, treated or untreated, in all of the anogenital areas. This is consistent with current regulatory requirements for EGWs treatments, as well as reflective of real life, as patients likely treat all of their warts and do not distinguish between baseline versus newly emerged warts in evaluating treatment success. Efficacy may also have been affected by the unexpectedly long duration of disease in the current studies, with *∼*70% of women having EGWs >6 months and most for more than a year. Longer duration of EGWs is associated with lower treatment clearance rates [25]. This may reflect, in part, selection for patients whose immune responses were inadequate to clear the warts on their own. 

Comparisons of the safety of imiquimod 3.75% with 5% may similarly be impacted by differences between the study designs, including usage of different AE coding systems due to changes in regulatory requirements. For the AEs reported by the most subjects, the incidences were generally comparable or lower for imiquimod 3.75% than for imiquimod 5% [21]. For example, the reported rates for application site pain were similar (*∼*8%) in women, while the rates for application site irritation/burning (5.5% versus 26%) and application site pruritus/itching (3.2% versus 32%) were lower for imiquimod 3.75% compared to imiquimod 5%. There was a slightly higher overall rate of discontinuations due to lost to follow-up in these studies as compared to the imiquimod 5% study [12]. The imiquimod 5% study, however, allowed for replacement of subjects discontinuing for nonsafety reasons, which may have reduced the overall discontinuation rate and the lost to follow-up rate. The similar proportions of women lost to follow-up within each treatment group in current studies, however, suggest these discontinuations were unlikely to be due to AEs or treatment failure. The lost to follow-up rates in the current studies, mostly immediately after the baseline visit, may reflect that EGWs are a sexually transmitted disease that tends to affect a younger, more mobile, and less compliant patient population. This highlights one of the major challenges of treatment in actual practice and emphasizes the importance of practitioner communication with the patient to encourage follow-up. The PP results in these studies may be more predictive than the ITT results of experience in clinical settings where patients are routinely receiving medical care, and therefore more likely to be adherent with treatment and follow-up. Direct comparative clinical studies of imiquimod 3.75% versus other treatment options for EGWs using the same endpoint of complete clearance of all EGWs, as well as safety assessments, may be helpful to assess relative efficacy and safety.

In conclusion, in a combined analysis in women treating EGWs for up to 8 weeks with imiquimod 2.5% and 3.75% creams applied daily, both imiquimod products were well tolerated and superior to placebo in completely clearing all baseline and newly emergent warts, as well as reducing wart counts compared to baseline. Imiquimod 3.75% cream, however, was also superior to 2.5% with respect to reducing wart counts and was the only product consistently superior to placebo in the analyses of the individual studies. The incremental efficacy of imiquimod 3.75% did not appear to be accompanied by an increase in intolerance. In women, the observed anatomic site-specific clearance rates were highest with imiquimod 3.75% cream in treating warts in the perineal and perianal areas. Imiquimod 3.75% cream applied daily is an EGW treatment option that may be more intuitive and reduce overall treatment duration for some women.

## Figures and Tables

**Table 1:** Demographic and external anogenital wart characteristics by treatment group, all women.

Women	Placebo	Imiquimod 2.5%	Imiquimod 3.75%	*P* value
	106	212	216	
Age, years				
Mean (standard deviation)	32.9 (12.2)	32.8 (11.6)	34.2 (13.1)	0.462^a^
Median (range)	29 (18–75)	30 (17–78)	31 (15–81)	

Race, *n* (%)				
White	71 (67.0)	132 (62.3)	157 (72.7)	0.107^b^
Black/African American	34 (32.1)	74 (34.9)	52 (24.1)	
Other	1 (0.9)	6 (2.8)	7 (3.2)	

Total wart area, mm^2^				
Mean (standard deviation)	166.5 (303.0)	161.9 (362.1)^c^	170.6 (463.0)	0.975^a^
Median (range)	57 (6–1969)^d^	55 (10–4000)^c^	50 (10–5579)	

Wart count, *n*				
Mean (standard deviation)	8.3 (7.7)	7.9 (6.3)^c^	7.8 (6.1)	0.751^a^
Median (range)	5 (2–30)	6 (2–30)^c^	6 (2–30)	

Duration since first EGW diagnosis, years				
Mean (standard deviation)	5.5 (8.3)	5.7 (7.8)	5.5 (8.1)	0.967^a^
Median (range)	1.4 (0.0–33.7)	2.1 (0.0–31.4)	1.6 (0.0–39.4)	

First episode, *n* (%)				
Yes	79 (74.5)	151 (71.2)	140 (64.8)	0.152^b^

Anatomical locations involved, *n* (%)				
1 only	54 (50.9)	93 (43.9)	96 (44.4)	0.451^b^
2 or more	52 (49.1)	119 (56.1)	120 (55.6)	
Vulvar	63 (59.4)^e^	138 (65.1)^e^	145 (67.1)^e^	
Inguinal	10 (9.4)^e^	30 (14.2)^e^	28 (13.0)^e^	
Perineal	51 (48.1)^e^	96 (45.3)^e^	109 (50.5)^e^	
Perianal	48 (45.3)^e^	103 (48.6)^e^	97 (44.9)^e^	
Perineal	51 (48.1)^e^	96 (45.3)^e^	109 (50.5)^e^	

^a^ *P* value from an ANOVA *F*test.

^b^ *P* value from a Chi-square test.

^c^One woman without wart data at baseline; wart count from screening used with no total area available.

^d^One woman with wart area <10 mm^2^.

^e^A woman may have more than one site involved, so total across anatomic sites may exceed 100%.

**Table 2:** Anatomic site-specific clearance rates, per protocol population.

Women	Placebo	Imiquimod 2.5%	Imiquimod 3.75%
	87	151	153
Anatomic site complete clearance, *n*/*N * ^a^ (%)			
Vulvar	14/51 (27.5)	42/98 (42.9)	53/104 (51.0)^b^
Inguinal	2/9 (22.2)	4/18 (22.2)	9/20 (45.0)
Perineal	11/43 (25.6)	36/68 (52.9)	48/74 (64.9)^b^
Perianal	10/40 (25.0)	34/69 (49.3)^b^	51/65 (78.5)^b,c^

Change in wart count from baseline, mean percent (standard deviation)			
Vulvar	−23.8 (56.4)	−52.3 (55.6)^d^	−56.8 (75.8)^d^
Inguinal	−27.2 (60.1)	−14.4 (69.5)	−49.3 (54.2)
Perineal	−21.3 (62.5)	−61.8 (46.1)^d^	−74.6 (42.9)^d^
Perianal	−11.6 (96.4)	−52.7 (59.5)^d^	−82.2 (40.4)^d,e^

^a^ *N *is women with anatomic site involved.

^b^Statistically significant versus placebo using Hochberg's modified Bonferroni procedure with *P* value determined by Cochran-Mantel-Haenszel test, stratified by analysis site two treatment groups at a time.

^c^Statistically significant versus imiquimod 2.5% using Hochberg's modified Bonferroni procedure with *P* value determined by Cochran-Mantel-Haenszel test, stratified by analysis site two treatment groups at a time.

^d^Statistically significant versus placebo using Hochberg's modified Bonferroni procedure with *P* value determined by ANCOVA with main effect treatment and controlling for baseline lesion count and analysis site.

^e^Statistically significant versus imiquimod 2.5% using Hochberg's modified Bonferroni procedure with *P* value determined ANCOVA with main effect treatment and controlling for baseline lesion count and analysis site.

**Table 3:** Summary of safety by treatment group, evaluation period, all women.

Women	Placebo	Imiquimod 2.5%	Imiquimod 3.75%
	106	211^a^	217^a^
Adverse events (AEs)^b^, *n* (%)			
Any AE^c^	39 (36.8)	88 (41.7)	85 (39.2)
Any serious	1 (0.9)	2 (0.9)	5 (2.3)
Any resulting in study discontinuation	1 (0.9)	3 (1.4)	5 (2.3)
Any severe grade	4 (3.8)	15 (7.1)	11 (5.1)
Any treatment-related AE^d^	4 (3.8)	41 (19.4)	42 (19.4)
Any resulting in study discontinuation	0 (0)	3 (1.4)	3 (1.4)
Application site reactions (ASRs), *n* (%)			
Any ASR^c^	4 (3.8)	39 (18.5)	38 (17.5)
Any severe	1 (0.9)	9 (4.3)	6 (2.8)

Treatment-related AEs in >1% women for imiquimod 3.75% that were more frequent than in placebo, *n* (%)^b,d^			
Application site pain	0 (0)	11 (5.2)	17 (7.8)
Application site irritation	1 (0.9)	11 (5.2)	12 (5.5)
Application site pruritus	2 (1.9)	14 (6.6)	7 (3.2)
Application site bleeding	1 (0.9)	1 (0.5)	3 (1.4)
Application site discharge	0 (0)	1 (0.5)	3 (1.4)
Application site erythema	0 (0)	3 (1.4)	3 (1.4)
Rest periods			
At least 1 rest period, *n* (%)	3 (2.8)	70 (33.2)	77 (35.5)
Dosing days missed due to rest, mean days (standard deviation)^e^	6.7 (4.7)	9.1 (7.2)	8.9 (7.2)

^a^One woman assigned to imiquimod 2.5% received 3.75%. Displayed here based on treatment received.

^b^Adverse events reported from start of treatment to 30 days after study.

^c^By preferred terms, regardless of causality assessment.

^d^Investigator assessed causality of related or probably related.

^e^For those women who took a rest.

**Table 4:** Summary of local skin reactions, maximum intensity during evaluation period.

	Placebo	Imiquimod 2.5%	Imiquimod 3.75%
Women	105^a^	193^a^	203^a^
Local skin reaction (LSR), *n* (%)			
Any LSR grade other than none	35 (33.3)	135 (69.9)	156 (76.8)
Severe grade			
Any	1 (1.0)	26 (13.5)	36 (17.7)
Erythema	0 (0)	18 (9.3)	20 (9.9)
Edema	0 (0)	5 (2.6)	4 (2.0)
Weeping/exudates	0 (0)	1 (0.5)	2 (1.0)
Flaking/scaling/dryness	0 (0)	2 (1.0)	0 (0)
Scabbing/crusting	0 (0)	0 (0)	1 (0.5)
Erosion/ulceration	1 (1.0)	17 (8.8)	26 (12.8)

^a^Denominator is the number of women with at least 1 postbaseline assessment.

## References

[1] Revzina NV, DiClemente RJ (2005). Prevalence and incidence of human papillomavirus infection in women in the USA: a systematic review. *International Journal of STD and AIDS*.

[2] Herzog TJ, Huh WK, Einstein MH (2010). How does public policy impact cervical screening and vaccination strategies?. *Gynecologic Oncology*.

[3] (2010). Sexually transmitted diseases treatment guidelines. Human papillomavirus (HPV) infection. *Morbidity and Mortality Weekly Report*.

[4] Koutsky L (1997). Epidemiology of genital human papillomavirus infection. *The American Journal of Medicine*.

[5] Dinh TH, Sternberg M, Dunne EF, Markowitz LE (2008). Genital warts among 18- to 59-year olds in the United States, national health and nutrition examination survey, 1999–2004. *Sexually Transmitted Diseases*.

[6] Woodhall SC, Jit M, Cai C (2009). Cost of treatment and QALYs lost due to genital warts: data for the economic evaluation of HPV vaccines in the United Kingdom. *Sexually Transmitted Diseases*.

[7] Pirotta M, Ung L, Stein A (2009). The psychosocial burden of human papillomavirus related disease and screening interventions. *Sexually Transmitted Infections*.

[8] Centers for Disease Control and Prevention (2009). *Sexually Transmitted Disease Surveillance, 2008*.

[9] Insinga RP, Dasbach EJ, Elbasha EH (2005). Assessing the annual economic burden of preventing and treating anogenital human papillomavirus-related disease in the US: analytic framework and review of the literature. *PharmacoEconomics*.

[10] Mayeaux EJ, Dunton C (2008). Modern management of external genital warts. *Journal of Lower Genital Tract Disease*.

[11] Gaspari AA (2007). Mechanism of action and other potential roles of an immune response modifier. *Cutis*.

[12] Edwards L, Ferenczy A, Eron L (1998). Self-administered topical 5% imiquimod cream for external anogenital warts. HPV Study Group. Human PapillomaVirus. *Archives of Dermatology*.

[13] Page KR, Sifakis F, Montes de Oca R (2006). Improved adherence and less toxicity with rifampin vs isoniazid for treatment of latent tuberculosis: a retrospective study. *Archives of Internal Medicine*.

[14] Gupta AK, Shear NH (2000). A risk-benefit assessment of the newer oral antifungal agents used to treat onychomycosis. *Drug Safety*.

[15] Farup PG (1992). Compliance with anti-ulcer medication during short term healing phase clinical trials. *Alimentary Pharmacology and Therapeutics*.

[16] Fife KH, Ferenczy A, Douglas JM (2001). Treatment of external genital warts in men using 5% imiquimod cream applied three times a week, once daily, twice daily, or three times a day. *Sexually Transmitted Diseases*.

[17] Gollnick H, Barasso R, Jappe U (2001). Safety and efficacy of imiquimod 5% cream in the treatment of penile genital warts in uncircumcised men when applied three times weekly or once per day. *International Journal of STD and AIDS*.

[18] Trofatter KF, Ferenczy A, Fife KH (2002). Increased frequency of dosing of imiquimod 5% cream in the treatment of external genital warts in women. *International Journal of Gynecology and Obstetrics*.

[19] Ferris DG, Baker D, Tyring S Imiquimod 2.5% and 3.75% applied daily for up to 8 weeks to treat external genital warts.

[20] Hochberg Y (1988). A sharper Bonferroni procedure for multiple tests of significance. *Biometrika*.

[21] (April 2009). Aldara (imiquimod cream, 5%) prescribing information.

[22] Data on file.

[23] Stockfleth E, Beti H, Orasan R (2008). Topical polyphenon E in the treatment of external genital and perianal warts: a randomized controlled trial. *The British Journal of Dermatology*.

[24] Stone KM, Becker TM, Hadgu A, Kraus SJ (1990). Treatment of external genital warts: a randomised clinical trial comparing podophyllin, cryotherapy and electrodesiccation. *Genitourinary Medicine*.

[25] Sauder DN, Skinner RB, Fox TL, Owens ML (2003). Topical imiquimod 5% cream as an effective treatment for external genital and perianal warts in different patient populations. *Sexually Transmitted Diseases*.

